# Sigmoid Volvulus and Ileosigmoid Knotting: An Update

**DOI:** 10.5152/eurasianjmed.2022.22310

**Published:** 2022-12-01

**Authors:** Sabri Selçuk Atamanalp, Rıfat Peksöz, Esra Dişçi

**Affiliations:** 1 Department of General Surgery, Atatürk University Faculty of Medicine, Erzurum, Turkey

**Keywords:** Sigmoid colon, Ileum, sigmoid volvulus, ileosigmoid knotting

## Abstract

Sigmoid volvulus and ileosigmoid knotting are uncommon intestinal obstructions, which generally affect adult males. The etiology is multifactorial. Volvulus triad including abdominal pain/tenderness, distention, and obstipation/constipation is the common clinical presentation. Although x-ray radiography helps with diagnosis, the current diagnostic procedure is computed tomography or magnetic resonance imaging in addition to flexible endoscopy in sigmoid volvulus. Endoscopic decompression is the primary treatment except for the presence of bowel gangrene and peritonitis in sigmoid volvulus, while such conditions and also ileosigmoid knotting require emergency surgery. The prognosis is relatively poor under these adverse circumstances and in ileosigmoid knotting.

**Main Points**

- Although sigmoid volvulus (SV) and ileosigmoid knotting (ISK) are uncommon intestinal obstruction forms, they have relatively wide geographic ranges. For this reason, some practitioners and some patients may encounter these nightmares someday.
- The current diagnostic procedure is computed tomography or magnetic resonance imaging in both diseases and additionally endoscopy in SV.
- Current management is flexible endoscopic decompression in SV, while complicated patients and those with ISK require emergency surgery.
- Despite modern diagnostic and therapeutic methods, the prognoses are still relatively poor, particularly in ISK.

## Introduction

**Figure 1. A-M.**
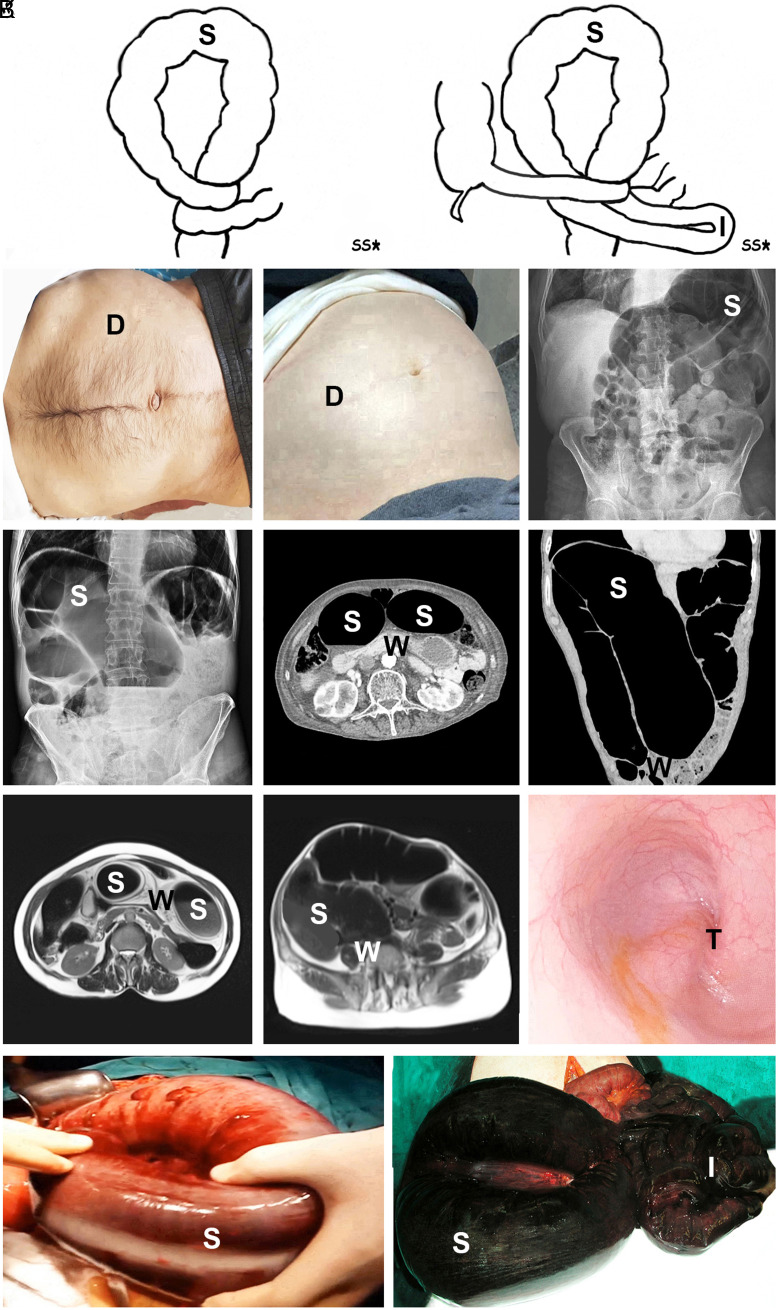
(A) Schematic diagram of SV. (B) Schematic diagram of ISK. (C) Abdominal appearance of SV. (D) Abdominal appearance of ISK. (E) Abdominal x-ray radiogram of SV. (F) Abdominal x-ray radiogram of ISK. (G) Abdominal axial CT image of SV. (H) Abdominal coronal CT image of ISK. (I) Abdominal axial MR image of SV. (J) Abdominal axial MR image of ISK. (K) Endoscopic appearance of SV. (L) Operative appearance of SV. (M) Operative appearance of ISK. CT, computed tomography; D, distention; I, ileum, ISK, ileosigmoid knotting; MR, magnetic resonance; S, sigmoid colon; SV, sigmoid volvulus T: torsioned lumen; W, whirl sign.

Sigmoid volvulus (SV), the rotation of the sigmoid colon around its base (Figure 1A), is a relatively rare intestinal obstruction form worldwide,^1-4^ while ileosigmoid knotting (ISK), the turning of the ileum or sigmoid colon around the other segment (Figure 1B), is extremely rare.^1,5-7^ However, both SV and ISK are relatively common in Eastern Anatolia.^8,9^ As a result of which, in our 56-year experience (from June 1966 to July 2022), the 1051-case SV series is the largest and the 80-case ISK series is the third largest monocenter patient serials in the world.^10,11^ In this review, based on the present comprehensive experience of Atatürk University Faculty of Medicine, the biggest health center of the region, an up-to-date information was offered in company with worldwide literature.

## Terminology

Sigmoid volvulus goes by the name of “common volvulus” due to its relative frequency when compared to ISK.^3^ Conversely, ISK is called as “rare,” “unusual,” or “unique volvulus.”^1,12-15^ Other names of ISK arising from its complex anatomic stricture are “compound,” “double-loop volvulus,” or “gordion knot.”^16-19^

## History

Sigmoid volvulus was first described by Rokitansky in 1836.^2,20,21^ Although Riverius mentioned some characteristics of ISK in the 16th century, it was also described by Rokitansky in 1836.^22,23^

## Epidemiology

Although SV has a wide geographic range, some regions including South America, Africa, Eastern and Northern Europe, Northern and Southern Asia, and the Middle East have high incidences of SV, which are known as “volvulus belts,” while North America, Western Europe, and Australia are low-incidence areas.^2,4,20^ Sigmoid volvulus constitutes 20%-50% of all intestinal obstructions in high-incidence regions, conversely, this rate is 3%-5% in the other areas.^2,4^ Our data demonstrate the incidence of SV as 18.8 patients per year and 4.2 patients per 100,000 persons per year. When our data are excluded, the unique study on this subject was reported from the United States, which presented this rate as 1.67 per 100,000 person-years.^2,24^ However, recent studies claim a relative decrease in SV incidence, most probably due to the westernization of dietary habits.^2,25-29^ On the other hand, ISK shows a similar worldwide distribution with a lower incidence consisting of 18%-27% of SV cases in high-incidence regions and 5%-8% in low-incidence areas.^6,7,9^ Hence, the total ISK cases reported to date is about 1000.^11,18^ According to our data, ISK comprises 7.1% of SV cases with an incidence of 1.4 patients per year and 0.3 patients per 100 000 persons per year.

Sigmoid volvulus frequently affects adults in the 4th-8th decades and the disease is more common in males with a ratio of 2/1 to 10/1,^1,2,4,25,27^ while ISK is generally seen in 3rd-5th decades with a male/female ratio of 2/1 to 6/1.^18^

## Etiology

A redundant sigmoid colon with an elongated and frequently narrow-based mesentery, dolichosigmoid, is the main anatomical prerequisite for SV.^30-34^ In ISK, additionally, a hypermobile terminal ileum is effective.^6,35^ Dolichosigmoid is rarely congenital as seen in childhood cases, whereas it is generally acquired.^2,36^ Despite some opposite ideas,^32^ according to general belief, advanced age increases dolichosigmoid, which results in increased SV and ISK incidences.^33-35,37,38^ Similarly, dolichosigmoid is more common in males; additionally, a relatively smaller pelvic inlet prevents derotation of the sigmoid colon causing both SV and ISK.^39^ In pregnancy, enlarged uterus plays a similar role and relatively increases the incidence of these entities.^40-43^

Due to undigested fiber, high-fiber and high-carbohydrate diets cause bulky stool, colonic fecal loading, and distention.^2,26,44^ Chronic constipation and some laxatives or enemas also lead to colonic distention.^2,26,45^ Living at high altitude induces the expansion of intracolonic gases (carbon dioxide, methane, and hydrogen) due to lower atmospheric pressure, which also results in the same problem.^44,46,47^ A similar result is seen in bad defecation habits as demonstrated in mentally retarded persons.^14,45,48^ Some neurologic entities such as Parkinson’s disease and Alzheimer’s disease trigger the same pathology by the way of neuronal destruction or used drugs.^1,44,49,50^ Various diseases including Hirschsprung’s disease or Chagas’ disease look like previously mentioned entities from the viewpoint of intestinal activity.^2,44,51^ In the end, chronic distention and increased intraluminal pressure worsen the elastogenesis of the colonic wall, and over time, dolichosigmoid occurs, which increases SV and ISK risks.^2,26,47^

## Pathophysiology

The sigmoid colon rotates from time to time and rotations less than 180° are considered to be physiological, which generally result in spontaneous derotation.^1,2,52,53^ However, untwisting requires much more force and a wider intraabdominal volume and it may be impossible due to the weariness of the sigmoid colon in addition to its enlargement arising from gas generation, which results in entrapment of the sigmoid colon and volvulus.^52,54^ Excessive torsions more than 180° frequently cause luminal obstruction, while vascular circulation is blocked when it passes 360°.^1,2,52,53^

Although dolichosigmoid is the principal condition in the development of SV and ISK, it does not occur in all risky people and all the time, because a triggering factor is generally needed.^35,52^ Acute diarrhea, sudden and excessive movements (reaping, harvesting, coitus, and delivery), and overeating following prolonged starvation (Ramadan fasting) are the main precipitators.^2,6,16,43,52,55-61^

In SV, due to the obstruction of the passage, the sigmoid colon enlarges, and additionally, fluid and electrolyte escape into the lumen. In ISK, this process is quicker and waxier due to the double-loop obstruction. Following the vascular blockage, ischemic injury occurs in the mucosa and it affects all layers in time. Bacterial translocation and absorption of toxic materials invoke shock. Increased intraabdominal volume pressure results in abdominal compartment syndrome.^2,16,18,20,24^

## Classification

Sigmoid volvulus is classified based on the clinical course (acute, subacute, or chronic), clinical severity (complete or incomplete), prevalence (sporadic or endemic), movement direction of the sigmoid colon (clockwise or counterclockwise), and volvulus degree of the sigmoid colon (180°, 360°, or ≥360°).^2,21^ Similarly, ISK has various classifications depending on prevalence (sporadic or endemic), volvulus direction of the sigmoid colon (clockwise or counterclockwise), and active bowel segment (ileum or sigmoid colon).^6,23^ However, none of these classifications procure any information about treatment options and prognosis in these diseases. By using age (mean life expectancy, 75 years in Turkey), American Society of Anesthesiologists physical status classification, bowel viability, and bowel anastomosis risk, Atamanalp^21,23^ described new updated classification systems including treatment algorithm and prognosis-estimating information for SV in 2020 (Table 1) and for ISK in 2021 (Table 2).

## Clinical Presentation

In SV, the most common symptoms and signs are abdominal pain/tenderness (65%-99%), generally asymmetrical left upper quadrant abdominal distention (88%-97%) (Figure 1C), and obstipation/constipation (52%-95%), which are passes for “volvulus triad” and encountered in 52%-93% of all cases. Other relatively rare clinical features are nausea/vomiting (40%-68%), hyperkinetic bowel sounds (30%-68%), empty rectum (42%-63%), hypokinetic/akinetic bowel sounds (31%-59%), fever (25%-28%), guarding/rebound tenderness (9%-15%), shock (7%-13%), and gangrenous stool (7%-11%).^1-4,20,24,25,28,56,58,62,63^

Ileosigmoid knotting generally has a quicker and waxier presentation with similar symptoms and signs including abdominal pain/tenderness (57%-100%), obstipation/constipation (59%-99%), and generally asymmetrical right upper quadrant abdominal distention (89%-97%) (Figure 1D), while volvulus triad is seen in 27%-100% of the cases. Other clinical features are nausea/vomiting (50%-78%), hypokinetic/akinetic bowel sounds (20%-63%), empty rectum (40%-60%), guarding/rebound tenderness (30%-48%), shock (28%-53%), hyperkinetic bowel sounds (10%-28%), and gangrenous stool (10%-15%).^5,7,9,18,12,19,22-24,62,64,65^

## Diagnosis

There is no pathognomonic routine laboratory test for SV and ISK. In SV, although various signs including omega or horseshoe, coffee bean, or bird beak signs have been described, plain abdominal x-ray radiographs are diagnostic in 57%-90% of the cases with a dilated sigmoid colon image generally in the left upper abdominal quadrant in addition to multiple small intestinal air-fluid levels (Figure 1E).^2,4,24,27,28,37,56,59,62,63,66^ In ISK, the unique difference is the localization of the above-mentioned sigmoid image generally in the right upper abdominal quadrant (Figure 1F) with an only 8%-10% of accuracy rate.^1,5,7-9,16-18,22,28,56,61,62,64,65,67^ Computed tomography (CT) or magnetic resonance imaging (MRI), the last which is generally preferred in pregnant, are highly diagnostic with 85%-98% of accuracy rates in both SV and ISK by demonstrating mesenteric whirl sign in addition to abovementioned sigmoid image (Figure 1G-J).^1,2,4,7,40,42,53,56,62-67^ Diagnostic endoscopy is also helpful in 75%-98% of SV cases by presenting a luminal twisting of the sigmoid colon lumen at a 20-30 cm distance from the anal verge (Figure 1K), while it remains incapable in the demonstration of ISK.^18,68^ Despite advanced techniques, 5%-10% of SV cases and 10%-30% of ISK cases are diagnosed at laparotomy with the abovementioned anatomical findings (Figure 1L and M).^2,4,7,18,66^ Misdiagnosis generally comprises nonspecific intestinal obstruction or acute abdominal emergency in SV, while additionally SV is a diagnostic mistake in ISK, and most of these cases require emergency action and surgery.^4,6,18,66^

## Treatment and Prognosis

In SV, following a rapid and effective resuscitation, endoscopic decompression is the primary treatment option with 55%-94% of success, 0%-2% of mortality, 2%-20% of morbidity, and 15%-55% of recurrence rates.^1,2,4,21,24,48,62,66,68-70^ For this purpose, rigid or preferably flexible endoscopes are used. Although flatus tubes are traditionally used following decompression, their recurrence preventive role is debated.^68^ Some selected nonelderly and well-conditioned patients are directed elective surgery consisting of sigmoid colectomy with 0%-2% of mortality, 5%-15% of morbidity, and 0%-1% of recurrence rates.^2,4,8,21^ In this field, laparoscopic procedure with natural orifice specimen extraction is the current option.^71^ On the other hand, patients with indefinite diagnosis, gangrenous stool, or peritoneal irritation findings during the admission in addition to unsuccessful endoscopic decompression are treated with emergency surgery. If the sigmoid colon is viable, surgical decompression alone (mortality 0%-5%, morbidity 5%-15%, and recurrence 15%-55%) may be performed, but a recurrence-reducing procedure such as sigmoidopexy, mesopexy, or mesoplasty (mortality 1%-10%, morbidity 10%-25%, and recurrence 10%-20%), or preferably sigmoid colectomy (mortality 1%-10%, morbidity 15%-25%, and recurrence 0%-1%) may also be added. Percutaneous endoscopic colopexy may be an alternative in elderly and bad-conditioned cases with 8%-15% of mortality, 13%-28% of morbidity, and 0%-15% of recurrence rates. In gangrenous cases, following the resection of gangrenous sigmoid colon, primary anastomosis (mortality 5%-10%, morbidity 10%-30%, and recurrence 0%-1%) is preferred in the restoration of the bowel continuity, while stoma is life-saving in elderly and bad-conditioned cases (mortality 20%-30%, morbidity 10%-30%, and recurrence 0%-1%).^1,2,4,8,21,24,27,38,58,59,62,66,68-70^

In ISK, emergency surgery following rapid and effective resuscitation is essential. In nongangrenous patients, decompression alone (mortality 1%-5% and morbidity 5%-15%) or in some selected nonelderly and well-conditioned patients, to prevent SV recurrence, sigmoid colopexy, mesopexy, or mesoplasty (mortality 1%-8% and morbidity 10%-20%) ore even sigmoid colectomy with primary anastomosis (mortality 1%-10% and morbidity 15%-25%) may be used. In patients with single-segment bowel gangrene, ileum or sigmoid colon resection with primary anastomosis (mortality 5%-20% and morbidity 10%-30%) or stoma (mortality 20%-50% and morbidity 30%-40%) is preferred, while patients with double-segment gangrene are treated with resection of both ileum and sigmoid colon in addition to double-segment primary anastomosis or ileum primary anastomosis and sigmoid stoma with 10%-60% of mortality and 20%-40% of morbidity rates.^5,7,9,17,18,22,23,24,28,38,56,61,62,64,67,72^

## Special Conditions

### Childhood

Sigmoid volvulus in childhood is a very rare clinical entity with a few 10 cases reported to date, whereas pediatric ISK is extremely rare declared little more than 20 patients. Diagnosis is more difficult due to the inability of both medical history and physical examination. Abdominal pain and distention are the main clinical features, while vomiting and diarrhea are more common when compared with that of adults. Computed tomography is the best diagnostic procedure, while endoscopic decompression by using flexible pediatric endoscopes is the primary treatment option in SV. The prognosis is worse than that of adults with 8%-40% and 15%-60% of mortality rates, respectively, while morbidity rates are 15%-50% and 20%-60%, respectively, in SV and ISK. Sigmoid volvulus tends to recur in early-onset SV or ISK, and for this reason, elective sigmoid colectomy is frequently recommended.^2,10,11,21,23,73^

### Pregnancy

Although SV is in the first 2 causes of intestinal obstruction in pregnancy, the total number is little more than 110 cases, whereas ISK is less often declared less than 20 patients. Clinical presentation of SV and ISK may sometimes be complex due to some physiologic features of pregnancy including nausea, vomiting, and abdominal pain. Although a single x-ray radiogram is allowed, flexible endoscopy and preferably MRI are the current diagnostic tools in SV, while MRI is the unique identifier in ISK. In SV, an enlarged uterus is thought as a preventer, even so, endoscopic decompression is the first treatment option. The management of these clinical entities requires a multidisciplinary approach and the prognoses are still relatively poor with 6%-15% of maternal and 20-30% of fetal mortality in addition to 20%-50% of morbidity in SV, whereas 15%-25% of maternal and 30%-50% of fetal mortality, additionally, 40%-60% of morbidity in ISK.^2,10,11,21,23,40-42,53,57, 60,74^

### Elderliness

About one-third of SV occurs in geriatric patients with a higher recurrence rate when compared with that of children and also adults. Endoscopic decompression is the first option in the management, whereas surgery has a relatively poor prognosis due to serious comorbidity, which consists of 15%-75% mortality with 20%-50% of morbidity in SV, and 20%-80% of mortality with 30%-60% of morbidity rates in ISK.^2,10,11,21,23,37-39,75^

## Figures and Tables

**Table 1.** Classification of Sigmoid Volvulus by Atamanalp^21^

Group	Definition	Treatment	Mortality (%)	Morbidity (%)	Recurrence (%)
1A	G0, A0, ASA1-3	Endoscopic decompression	0-1	1-2	15-55
		Or plus elective surgical resection and anastomosis	0-2	5-15	0-1
1B	G0, A1 or ASA4-5	Endoscopic decompression	5-10	10-25	15-55
		Or plus percutaneous endoscopic colopexy	8-15	15-30	0-15
		Or plus elective percutaneous endoscopic colopexy	5-13	13-28	0-15
2A	G0, A0, ASA1-3, E1	Surgical decompression	1-5	5-15	15-55
		Or plus surgical colopexy or mesopexy or mesoplasty	1-8	10-20	10-20
		Or plus surgical resection and anastomosis	1-10	15-25	0-1
2B	G0, A1, or ASA4-5, E1	Surgical decompression	10-30	20-40	15-55
3A	G1, A0, ASA1-3, B0	Surgical resection and anastomosis	5-10	10-30	0-1
3B	G1, A1, or ASA4-5 or B1	Surgical resection and stoma	20-30	30-60	0-1

A0, age < 75 years; A1, age ≥ 75; ASA1, no other disease; ASA2, mild systematic disease; ASA3, severe systematic disease; ASA4, life-threatening systematic disease; ASA5, moribund; B0, normal anastomotic risk; B1, increased anastomotic risk (ischemia, edema, perforation, or different diameter); G0, viable bowel; G1, gangrenous bowel.

**Table 2.** Classification of Ileosigmoid Knotting by Atamanalp^23^

Group	Definition	Surgical Treatment	Mortality (%)	Morbidity (%)
1A	G0, A0, ASA1-3	Decompression	1-5	5-15
		Or plus colopexy or mesopexy or mesoplasty	1-8	10-20
		Or plus sigmoid resection and anastomosis	1-10	15-25
1B	G0, A1 or ASA4-5	Decompression	10-30	20-40
2A	G1, A0, ASA1-3, B0	Ileum or sigmoid colon resection and anastomosis	5-20	10-30
2B	G1, A1 or ASA4-5 or B1	Ileum or sigmoid colon resection and stoma	20-50	30-60
3A	G2, A0, ASA1-3, B0	Ileum and sigmoid colon resection and anastomosis	10-30	20-40
3B	G2, A1 or ASA4-5 or B1	Ileum and sigmoid colon resection, one anastomosis, and one stoma	30-60	40-80

A0, age < 75 years; A1, age ≥ 75; ASA1, no other disease; ASA2, mild systematic disease; ASA3, severe systematic disease; ASA4, life-threatening systematic disease, ASA5, moribund, B0, normal anastomotic risk, B1, increased anastomotic risk (ischemia, edema, perforation, or different diameter); G0, viable bowel; G1, gangrenous ileum or sigmoid colon; G2, gangrenous ileum and sigmoid colon.

## References

[1] Bauman ZM Evans CH . Volvulus. Surg Clin North Am. 2018;98(5):973 993. (10.1016/j.suc.2018.06.005) 30243456

[2] Raveenthiran V Madiba TE Atamanalp SS De U . Volvulus of the sigmoid colon. Colorectal Dis. 2010;12(7 Online):e1 e17. (10.1111/j.1463-1318.2010.02262.x) 20236153

[3] Williams W Sigmoid volvulus: a common cause of bowel obstruction. US Paharmacist. 2020;45(12):HS12 HS16.

[4] Swenson BR Kwaan MR Burkart NE et al. Colonic volvulus: presentation and management in metropolitan Minnesota, United States. Dis Colon Rectum. 2012;55(4):444 449. (10.1097/DCR.0b013e3182404b3d) 22426269

[5] Mandal A Chandel V Baig S . Ileosigmoid knot. Indian J Surg. 2012;74(2):136 142. (10.1007/s12262-011-0346-y) 23542502 PMC3309095

[6] Alver O Oren D Tireli M Kayabaşi B Akdemir D . Ileosigmoid knotting in Turkey – review of 68 cases. Dis Colon Rectum. 1993;36(12):1139 1147. (10.1007/BF02052263) 8253011

[7] Gupta AK Ansari MAM Jayant S Goel S Bansal LK . Ileosigmoid knotting causing double-lumen acute intestinal obstruction and gangrene – review and a case report. J Clin Diagn Res. 2020;14(10):PE06 PE11. (10.7860/JCDR/2020/45118.14130)

[8] Atamanalp SS Atamanalp RS . Sigmoid volvulus: avoiding recurrence. Tech Coloproctol. 2019;23(4):405 406. (10.1007/s10151-019-01984-1) 30955105

[9] Atamanalp SS Oren D Başoğlu M et al. Ileosigmoidal knotting: outcome in 63 patients. Dis Colon Rectum. 2004;47(6):906 910. (10.1007/s10350-004-0528-9) 15129310

[10] Web of Science. Sigmoid volvulus. Available at: https://www.webofscience.com/wos/woscc/summary/19339455-5b16-48a9-8da7-4aa71ee7152f-6575a70b/recently-added/1. Accessed December 14, 2022.

[11] Web of Science. Ileosigmoid knotting. Available at: https://www.webofscience.com/wos/woscc/summary/1a283576-3a8e-4e54-8c44-2d0b7c2c6827-65759828/recently-added/1. Accessed December 14, 2022.

[12] Sangwan M Sangwan V Garg MK Mutreja J Singla D Gautam D . Ileosigmoid knotting: a rare case report with review of literature. J Surg Case Rep. 2015;2015(5):1 3. (10.1093/jscr/rjv051) PMC4429256 25972410

[13] Rahimi-Movaghar E Tahouri T . Ileo-sigmoid knotting – an unusual cause of intestinal obstruction. A case report. Int J Surg Case Rep. 2022;98:107511. (10.1016/j.ijscr.2022.107511) PMC9418438 35985117

[14] Boussaidane S Samlali A Hamri A Narjis Y Benelkhaiat BR . Ileosigmoid knot in a patient with Down syndrome: a unique surgical emergency. Pan Afr Med J. 2021;38(8):8. (10.11604/pamj.2021.38.8.27407) 33520077 PMC7825375

[15] Bain K Lelchuk A Nicoara M Meytes V . A unique surgical emergency: ileosigmoid knotting. AME Case Rep. 2018;2:29. (10.21037/acr.2018.06.02) PMC6155566 30264025

[16] Mba EL Obiano SK Mshelia NM . Compound volvulus: a case report and literature review. J Surg Case Rep. 2018;11:1 3. 10.1093/jscr/rjy311 PMC6232282 30443321

[17] Gibney EJ Mock CN . Ileosigmoid knotting. Dis Colon Rectum. 1993;36(9):855 857. (10.1007/BF02047383) 8375228

[18] Atamanalp SS Disci E Peksoz R Atamanalp RS Atamanalp CT . Ileosigmoid knotting: a review of 923 cases. Pak J Med Sci. 2022;38(3Part-I):711 715. (10.12669/pjms.38.3.5320) 35480527 PMC9002437

[19] Bhambare M Waghmare S Tiwari A Pandya J . Ileosigmoid knotting – a disastrous double closed loop obstruction. Int J Surg Case Rep. 2014;5(12):1035 1037. (10.1016/j.ijscr.2014.09.037) 25460467 PMC4276070

[20] Ballantyne GH Review of sigmoid volvulus. Clinical patterns and pathogenesis. Dis Colon Rectum. 1982;25(8):823 830. (10.1007/BF02553326) 6293790

[21] Atamanalp SS Sigmoid volvulus: an update for Atamanalp classification. Pak J Med Sci. 2020;36(5):1137 1139. (10.12669/pjms.36.5.2320) 32704301 PMC7372645

[22] Vaez-Zadeh K Dutz W . Ileosigmoid knotting. Ann Surg. 1970;172(6):1027 1033. (10.1097/00000658-197012000-00016) 5496474 PMC1397161

[23] Atamanalp SS Ileosigmoid knotting: an update for Atamanalp classification. Pak J Med Sci. 2021;37(3):913 915. (10.12669/pjms.37.3.3179) 34104188 PMC8155425

[24] Ballantyne GH Brandner MD Beart RW Ilstrup DM . Volvulus of the colon. Incidence and mortality. Ann Surg. 1985;202(1):83 92. (10.1097/00000658-198507000-00014) 4015215 PMC1250842

[25] Atamanalp SS Disci E Atamanalp RS . Sigmoid volvulus: what has changed in the last 50 years? Ethiop Med J. 2017;55(4):279 284.

[26] Karatas N Atamanalp SS . Sigmoid volvulus: dietary and defecation habits. Jokull. 2021;71(2):19 23.

[27] Heis HA Bani-Hani KE Rabadi DK et al. Sigmoid volvulus in the Middle East. World J Surg. 2008;32(3):459 464. (10.1007/s00268-007-9353-3) 18196324

[28] Gibney EJ Colonic volvulus. Dis Colon Rectum. 1989;32(12):1080. (10.1007/BF02553888) 2591284

[29] Segal I Decreasing incidence of sigmoid volvulus at Baragwanath Hospital Johannesburg. S Afr Med J. 1985;67(12):443. 3983725

[30] Madiba TE Haffajee MR . Sigmoid colon morphology in the population groups of Durban, South Africa, with special reference to sigmoid volvulus. Clin Anat. 2011;24(4):441 453. (10.1002/ca.21100) 21480385

[31] Akinkuotu A Samuel JC Msiska N Mvula C Charles AG . The role of the anatomy of the sigmoid colon in developing sigmoid volvulus. A case-control study. Clin Anat. 2011;24(5):634 637. (10.1002/ca.21131) 21322064 PMC3291329

[32] Bhatnagar BNS Sharma CLN Gupta SN Mathur MM Reddy DCS . Study on the anatomical dimensions of the human sigmoid colon. Clin Anat. 2004;17(3):236 243. (10.1002/ca.10204) 15042573

[33] Bayeh AB Abegaz BA . The role of sigmoid colon dimensions in the development of sigmoid volvulus, North-Western Ethiopia. PLoS One. 2021;16(12):e0260708. (10.1371/journal.pone.0260708) PMC8635388 34851992

[34] Sadahiro S Ohmura T Yamada Y Saito T Taki Y . Analysis of length and surface area of each segment according to age, sex, and physique. Surg Radiol Anat. 1992;14(3):251 257. (10.1007/BF01794949) 1440190

[35] Korkut E Atamanalp SS . Factors triggering knot formation in ileosigmoid knotting. Pak J Med Sci. 2022;38(6):1714 1716. (10.12669/pjms.38.6.6133) 35991231 PMC9378406

[36] Raahave D Dolichocolon revisited: an inborn anatomic variant with redundencies causing constipation and volvulus. World J Gastrointest Surg. 2018;10(2):6 12. (10.4240/wjgs.v10.i2.6) 29492185 PMC5827035

[37] Bak MP Boley SJ . Sigmoid volvulus in elderly patients. Am J Surg. 1986;151(1):71 75. (10.1016/0002-9610(86)90014-0) 3946751

[38] Singh PK Ali MS Manohar DB Sethi M . A challenging case of ileosigmoid knotting in an elderly. Cureus. 2020;12(8):e9624. (10.7759/cureus.9624) PMC7478921 32923225

[39] Avots-Avotins KV Waugh DE . Colon volvulus and the geriatric patient. Surg Clin North Am. 1982;62(2):249 260. (10.1016/s0039-6109(16)42684-8) 7071692

[40] Atamanalp SS Öztürk G . Sigmoid volvulus in pregnancy. Turk J Med Sci. 2012;42(1):9 15. (10.3906/sag-1101-2)

[41] Serafeimidis C Waqainabete I Creaton A Vakamacawai E Kumar R . Sigmoid volvulus in pregnancy: case report and review of literature. Clin Case Rep. 2016;4(8):759 761. (10.1002/ccr3.617) 27525078 PMC4974422

[42] Atamanalp SS Ileosigmoid knotting in pregnancy. Turk J Med Sci. 2012;42(4):553 558. (10.3906/sag-1108-41)

[43] Abebe E Suga Y Tadese M Tesfaye T . Ileosigmoid knotting in grand multipara women during labor. A rare occurence. Ethiop Med J. 2017;55(4):313 316.

[44] Asbun HJ Castellanos H Balderrama B et al. Sigmoid volvulus in the high altitude of the Andes. Review of 230 cases. Dis Colon Rectum. 1992;35(4):350 353. (10.1007/BF02048112) 1582356

[45] Atamanalp SS Sigmoid volvulus: relationship between mental retardation. Gazi Med J. 2021;32(4):32.

[46] Borda Mederos LA Kcam Mayorca EJ Alarcon Aguilar P Miranda Rosales LM . Andean megacolon and sigmoid volvulus in the high altitude. Presentation of 418 cases between 2008-2012 at C. Monge Hospital, Puno, Peru. Rev Gastrointest Peru. 2017;37(4):317 322. 29459800

[47] Anand AC Sashindran VK Mohan L . Gastrointestinal problems at high altitude. Trop Gastroenterol. 2006;27(4):147 153. 17542291

[48] da Rocha MC Capela T Silva MJ Ramos G . Endoscopic management of sigmoid volvulus in a debilitated population: what relevance? GE Port J Gastroenterol. 2020;27(3):160 165. 32509921 10.1159/000504721 PMC7250355

[49] Tateno F Sakakibara R Aiba Y Ogata T Katsumata M Matsuoka Y . Recurrent sigmoid volvulus in a patient with Parkinson’s disease. Clin Auton Res. 2020;30(3):283 285. (10.1007/s10286-019-00658-0) 31838591

[50] Blackley S Maguire C Daniels T . Seven cases of sigmoid volvulus in Parkinson’s disease. J R Coll Physicians Edinb. 2016;46(3):157 159. (10.4997/JRCPE.2016.303) 27959348

[51] Uylas U Gunes O Kayaalp C . Hirschsprung’s disease complicated by sigmoid volvulus: a systematic review. Balk Med J. 2021;38(1):1 6. (10.4274/balkanmedj.galenos.2020.2020.4.131) PMC8909226 32856883

[52] Dişçi E Atamanalp SS . Factors precipitating volvulus formation in sigmoid volvulus. Ulus Travma Acil Cerrahi Derg. 2022;28(3):281 284. (10.14744/tjtes.2020.03762) 35485550 PMC10493538

[53] Perrot L Fohlen A Alves A Lubrano J . Management of colonic volvulus. J Visc Surg. 2016;153(3):183 192. (10.1016/j.jviscsurg.2016.03.006) 27132752

[54] Dahlberg M Hallqvist Everhov Å . Re: Entrapment is an essential feature of sigmoid volvulus. ANZ J Surg. 2020;90(9):1823 1824. (10.1111/ans.16053) 32924301

[55] Koc C Uylas U Kayaalp C . Sigmoid volvulus provoked by severe diarrhea. Gazi Med J. 2020;31(2):204 205.

[56] Chalya PL Mabula JB . Sigmoid volvulus and ileo-sigmoid knotting: a five year experience at a tertiary care hospital in Tanzania. World J Emerg Surg. 2015;10:10. (10.1186/s13017-015-0001-1) PMC4359572 25774209

[57] Ucar NS Yuksel BC Hengirmen S . Did ileal knotting trigger labor or did labor cause ileal knotting? Report of a case. Surg Today. 2009;39(5):440 443. (10.1007/s00595-008-3850-3) 19408085

[58] Saidi F The high incidence of intestinal volvulus in Iran. Gut. 1969;10(10):838 841. (10.1136/gut.10.10.838) 5350109 PMC1552983

[59] De U Sigmoid volvulus in rural Bengal. Trop Doct. 2002;32(2):80 82. (10.1177/004947550203200207) 11931206

[60] Boukhalit H Zamani O Jroundi L . A case of ileosigmoid knotting in grand multipara in the postpartum period. Pan Afr Med J. 2019;32(1):106. 10.11604/pamj.2019.32.106.13542 PMC6561003 31223396

[61] Banerjee C Mukhopadhyay M Roy A Kumar J Mukherjee S Rahman QM . The unusual volvulus: a five year retrospective analysis of nine cases. Indian J Surg. 2014;76(2):100 103. (10.1007/s12262-012-0551-3) 24891772 PMC4039685

[62] Kapadia MR Volvulus of the small bowel and colon. Clin Colon Rect Surg. 2017;30(1):40 45. (10.1055/s-0036-1593428) PMC5179272 28144211

[63] Atamanalp SS Sigmoid volvulus: diagnosis in 938 patients over 45.5 years. Tech Coloproctol. 2013;17(4):419 424. (10.1007/s10151-012-0953-z) 23224856

[64] Ooko PB Saruni S Oloo M Topazian HM White R . Ileo-sigmoid knotting: a review of 61 cases in Kenya. Pan Afr Med J. 2016;23:198. (10.11604/pamj.2016.23.198.6255) PMC4907762 27347287

[65] Mbanje C Mungazi SG Muchuweti D Mazingi D Mlotshwa M Maunganidze AJV . Ileo-sigmoid knotting: the Parirenyatwa Hospital experience. S Afr J Surg. 2020;58(2):70 73. (10.17159/2078-5151/2020/v58n2a3174) 32644309

[66] Lai SH Vogel JD . Diagnosis and management of colonic volvulus. Dis Colon Rectum. 2021;64(4):375 378. (10.1097/DCR.0000000000001947) 33496483

[67] Atamanalp SS Ileosigmoid knotting: one of the largest single-center series. Pak J Med Sci. 2018;34(3):671 675. (10.12669/pjms.343.14893) PMC6041517 30034437

[68] Atamanalp SS Endoscopic decompression of sigmoid volvulus: review of 748 patients. J Laparoendosc Adv Surg Tech A. 2022;32(7):763 767. (10.1089/lap.2021.0613) 34748412

[69] Alavi K Poylin V Davids JS et al. The American Society of Colon and Rectal Surgeons clinical practice guidelines for the management of colonic volvulus and acute colonic pseudo-obstruction. Dis Colon Rectum. 2021;64(9):1046 1057. (10.1097/DCR.0000000000002159) 34016826

[70] Abdelrahim A Zeidan S Qulaghassi M Ali O Boshnaq M . Dilemma of sigmoid volvulus management. Ann R Coll Surg Engl. 2022;104(2):95 99. (10.1308/rcsann.2021.0123) 34860119 PMC10335211

[71] Nguyen SH Tavares K Chinn A Russell D Gillern S Yheulon C . Is laparoscopy underutilized for sigmoid volvulus? Surg Laparosc Endosc Percutan Tech. 2022;32(5):564 570. (10.1097/SLE.0000000000001074) 35960695

[72] Atamanalp SS Treatment for ileosigmoid knotting: a single-center experience of 74 patients. Tech Coloproctol. 2014;18(3):233 237. (10.1007/s10151-013-1046-3) 23839796

[73] Damkjaer MB Farooqui W Ifaoui I Penninga L . Sigmoid volvulus in children. BMJ Case Rep. 2021;14(5):e241869. (10.1136/bcr-2021-241869) PMC8117995 33980558

[74] Watanabe T Kinjo T Kinjyo Y et al. Sigmoid volvulus in pregnancy assessed by contrast-enhanced computed tomography scanning. Case Rep Obstet Gynecol. 2021;2021:6692483. (10.1155/2021/6692483) PMC7952176 33747587

[75] Machado NO Ileosigmoid knot: a case report and literature review of 280 cases. Ann Saudi Med. 2009;29(5):402 406. (10.4103/0256-4947.55173) 19700901 PMC3290047

